# ANO1 inhibits cardiac fibrosis after myocardial infraction via TGF-β/smad3 pathway

**DOI:** 10.1038/s41598-017-02585-4

**Published:** 2017-05-24

**Authors:** Yao Gao, Yan Mei Zhang, Li Jun Qian, Ming Chu, Jian Hong, Di Xu

**Affiliations:** Department of Geriatrics, the First Affiliated Hospital of Nanjing Medical University, Nanjing, 210029 China

## Abstract

As a newly identified factor in calcium-activated chloride channel, ANO1 participates in various physiological processes like proliferation and differentiation, and expresses in human cardiac fibroblasts. In this experiment, we investigated the function of ANO1 in cardiac fibrosis after myocardial infraction (MI) with methods of Western blotting, Quantitative real-time PCR (qRT-PCR), metabolic reduction of 3-(4,5-dimethylthiozol-2-yl)-2, 5-diphenyltetrazo-lium bromide (MTT), immunofluorescence and confocal imaging, and Masson’s trichrome staining. The results showed that the expression of ANO1 significantly increased in neonatal rats’ cardiac fibroblasts after hypoxia and in cardiac tissues after MI. After ANO1 over-expression, cardiac fibrosis was reduced *in vitro* and *in vivo*. Moreover, the expression of TGF-β and p-smad3 declined after ANO1over-expression in cardiac fiborblasts. In conclusion, ANO1 inhibits cardiac fibrosis after MI via TGF-β/smad3 pathway in rats.

## Introduction

Myocardial infarction(MI) is a leading cause of death worldwide^1^. Although percutaneous coronary intervention (PCI) has evidently improved the survival, post-MI heart failure still occurs after various adverse cardiac remodeling, including cardiac fibrosis, cardiomyocyte apoptosis, inflammatory reaction, etc^2^. Cardiac fibrosis, characterized by an excessive accumulation of extracellular matrix (ECM), is a process underlying cardiac remodeling post-MI^3^. Myocardium consists of cardiac fibroblasts (CFs), cardiomyocytes, smooth muscle cells, and endothelial cells. Cardiac fibroblasts, making up more than 90% of the non-myocytes, play a major role in normal cardiac function and cardiac fibrosis^4–6^. In typical cardiac fibrosis, exessive proliferation of fibroblasts and deposition of extracellular matrix (ECM) proteins are stimulated by cardiac fibroblasts in the myocardium^7–9^. Cardiac fibrosis is a common pathological hallmark of many heart diseases including acute and chronic cardiovascular disorders, and contributes to systolic and diastolic dysfunction in many cases^10^. However, effective therapies for cardiac fibrosis remain undeveloped.

Calcium-activated Chloride Channels (CaCCs) in almost all tissues regulate smooth muscle excitability, epithelial fluid secretion, signal transduction, nociception, cell proliferation and other physiological processes^11, 12^. In 2008, three research groups identify that ANO1 (anoctamin-1, also known as Transmembrane protein 16A (TMEM16A) (Oral cancer overexpressed 2 (ORAOV2), discovered on gastroin- testinal stromal tumor 1 (DOG1)or tumor-amplified and overexpressed sequence 2 (TAOS2))located on human chromosome 11q13 is a key constituent of CaCCs^13–15^. The anoctamin family has 10 members including ANO1-10 (TMEM16A-K). *Ano1* gene contains 26 exons encoding a 960 amino acid protein that is composed of eight transmembrane domains (TMs) and one pore-loop between TM5 and TM6, and ANO1 follows the biophysical properties of CaCCs including motility, attachment, and cell proliferation^13, 16, 17^. ANO1 is also involved in tumorigenesis, like gastrointestinal stromal tumors, oral cancers, head and neck squamous cell carcinomas, breast cancers, prostate cancers, and glioblastomas^17–23^. Antoun EI Chemaly and his colleagues verifies the presence of ANO1 in human atria fibroblasts^24^.

Transforming growth factor beta (TGF-β) is a cytokine regulating cell apoptosis, proliferation, and ECM production^25–27^. In mammals, there are three types of TGF-β: TGF-β1, TGF-β2, and TGF-β3. TGF-βbound to receptors of TGF-βphosphorylate downstream targets of Smad (homologues of mothers against decapentaplegic in *Drosophila* and sma-2, -3, and -4 in *Caenorhabditis elegans*) protein 2 and 3 at serine residues^28, 29^. In 2009, M.A.Olman announced that TGF- β/Smad3 pathway induces myofibroblasts expression and enhances deposition of extracellular matrix proteins such as collagen I and III via Smad3 activation^30^.

Based on these findings, we propose that ANO1 takes part in the process of cardiac fibrosis via TGF-β/Smad3 signaling pathway.

## Materials and Methods

### Antibodies

Primary antibodies: TMEM16A antibody (Santa Cruz Biotechnology, Inc., Texas, USA), α-smooth muscle actin (α-SMA) antibody and TGF-β1 antibody (Epitomics, Inc., California, USA), Smad3 antibody and Smad3 (phospho S423+S425) antibody (Abcam, Inc., Cambridge,UK), Vimentin antibody(Abcam, Inc., Cambridge,UK), and Collagen I antibody (wanleibio co.,ltd, Shenyang, China), and Gapdh antibody (HuaAnBiotech, Inc., Hangzhou, China).

Secondary antibodies: horseradish peroxidase-conjugated rabbit-anti-goat IgG antibody (HuaAnBiotech, Inc., Hangzhou, China), peroxidase-conjugated affinipure goat anti-rabbit IgG (H+L) (Jackson ImmunoResearch Laboratories, Inc., PA, USA), Alexa Fluor ®488-conjugated affinipure goat anti-rabbit lgG (H+L), Cy^TM^ 3-conjugated affinipure goat anti- mouse^++^ lgG (H+L), and Alexa Fluor ®488-conjugated affinipure donkey anti-goat++ lgG (H+L) (Jackson ImmunoResearch Laboratories, Inc., PA, USA).

### Cardiac fibroblast isolation and culture

Neonatal rat cardiac fibroblasts were isolated from 1–3-day-old Sprague–Dawley rats (Nanjing Medical University Laboratory Animal Center, Nanjing, China) as previously described^31^. Cardiac fibroblasts were cultured in 10-cm FALCON polystyrene dishes (Corning, NY, USA) at 37 °C with 5% CO_2_, supplemented with High Glucose Dulbecco’s Modified Eagle’s Medium (DMEM, GIBCO, Inc., USA), 10% fetal calf serum (PAA, Dartmouth, MA), and antibiotics (penicillin and streptomycinat). The third passage cardiac fibroblasts were used in the following experiments.

### Cardiac fibroblasts hypoxia

After serum starvation, cardiac fibroblasts were incubatedin a GENbag anaer (bioMerieux® sa, Marcy I’Etoile, France) at 37 °C with 5% CO_2_, 1% O_2_ and 94% N_2_ for 6, 8, 10, 16, and 24 hours.

### Cardiac fibroblast transfection

The adenovirus vector with green fluorescent protein (GFP) labeled with *Ano1* gene (Ad-ANO1-GFP) to up-regulate ANO1 expression, was constructed by Shanghai Jikai Gene Technology Co., Ltd. As a negative control (NC), adenovirus vector labeled with green fluorescence protein (Ad-GFP) was to figure out the optimal transfection concentration for this study. The optimal efficiency of infection was determined by the rate of GFP expression and the cell viability. Briefly, reconstructed adenovirus (stored at −80 °C) with an original concentration of 6*10^10^ plaque-forming units/ml (PFU/ml) was diluted for 50 times in enhanced infection solution (stored at −20 °C). Ad-GFP of different concentration (5*10^6^ PFU/ml, 5*10^7^ PFU/ml, and 5*10^8^ PFU/ml) was respectively transferred into cardiac fibroblasts within the third generation in DMEM without serum. After adenovirus transfection (24 hours), the cardiac fibroblasts were cultured for 24 hours with complete medium. Then the cell growth and green fluorescence protein (GFP) expression were observed with inversion fluorescence microscope. We chose the best multiplicity of infection according to the rate of GFP expression and the cell viability. The optimal transfection concentration was determined and used in the following experiments.

The third generation of cardiac fibroblasts were randomly divided into three groups and transfected with Ad-GFP or Ad-ANO1-GFP using the optimal transfection concentration: a. control group; b. Ad-GFP group; c. Ad-ANO1-GFP group.

### Animal model of MI and gene transfer *in vivo*

C57Bl/6J male mice, age of 8–10 weeks, were used for the experiments. Mice were divided into four groups by random: sham, MI, Ad-GFP+MI and Ad-ANO1+MI. The left anterior descending coronary artery (LAD) was ligated to induce MI^32^. Briefly, mice were anesthetized with 1% sodium pentobarbital and then intubated and ventilated during the operative process. The LAD was ligated about 2mmfrom the tip of the left auricle with 7–0 silk suture in MI group. The same procedure was performed in sham group without the ligation of the suture under the LAD.MI was confirmed by the elevation of S-T segment on an electrocardiogram. Mice in Ad-GFP+MI and Ad-ANO1+MI were given intramyocardial injection of 45 ul Ad-GFP or Ad- *Ano1* (1*10^10^ pfu/ml) into the left ventricular wall bordering the infarction zone via a 30-gauge Hamilton needle, while mice in the sham and MI group received the same amount of saline. The animals were sacrificed 1 week after surgery for further analysis.

The research was approved by the ethical committee of Nanjing Medical University and all animal experiments were performed in compliance with the guidelines on humane use and care of laboratory animals for biomedical research published by National Institutes of Health (No. 85-23, revised 1996).

### Masson’s trichrome staining

The hearts were collected, fixed in 4% buffered formalin, embedded in paraffin, and cut into 5-um sections. Masson’s trichrome staining was performed to analyze fibrosis according to previously described methods^33^.

### Western blotting

Cardiac fibroblasts were collected in cold buffer and the protein extracts were obtained as previously described^31^. The left ventricular tissues were lysed using RIPA buffer containing a protease inhibitor cocktail. The lysates were centrifuged at 12,000 g for 20 min (4 °C) and the supernatants were collected. Equal amounts of protein (30 μg) was separated by 10% SDS-PAGE and transferred to Polyvinylidene Fluoride (PVDF) membranes(Millipore, Inc., Massachusetts, USA). The membranes were incubated in 5% Bull Serum Albumin (BSA) at room temperature for 1 hour, and then incubated with the following primary antibodies: TMEM16A, α-SMA, TGF-β1, Smad3, Smad3 (phospho S423+425), Collagen I, and GAPDH antibodies at 4 °C for 12 hours. Next, we used peroxidase-conjugated goat anti-rabbit IgG and anti-mouse IgG secondary antibody to incubate the PVDF membranes at 4 °C for 2.5 h, and usedan hypersensitive chemiluminescence kit (wanleibio co.,ltd, Shenyang, China) to detect the expression of these proteins.

### Quantitative real-time PCR (qRT-PCR)

Total RNA was extracted from PA samples with TRIzol Reagent (life technologies,USA).Gene-specific primers were used to amplify *Tmem16a* (5_-GAAAACCATCAACTCGGTTCTGC-3_ and 5_-GTCGAATAGGTGTTGCTTCTCC-3_) and GAPDH (5_-GGCCTTCCGTGTTCC-3_ and 5_-CGCCTGCTTCACCACCTTC-3_). The extracted RNA was reverse-transcribed into cDNA with the PrimeScript™ RT Master Mix (TaKara), and qRT-PCR was carried out using the SYBR Premix Ex Taq™ II (TaKara), with GAPDH (KGDN20-R)as the internal control. All the qRT-PCR analyses were performed on an Applied Biosystems StepOnePlus Real-Time PCR System, according to the protocol provided by the manufacturer.

### MTT assay for cell viability

Cell viability was evaluated using a colorimetric method based on the metabolic reduction of 3-(4, 5-dimethylthiozol-2-yl)-2, 5-diphenyltetrazo-lium bromide (MTT) dye to formazan, as previously described^31^. Briefly, cardiac fibroblasts were plated onto 96-multiwell plates, 8,000 cells per well. Another 24 hours later, cells cultured under hypoxic conditions at the point of 6, 8, 10, 16 and 24 hours were rinsed with phosphate buffer saline (PBS), and then MTT was added. Then, 4 hours later, dimethyl sulfoxide (DMSO, Sigma-Aldrich, Inc., USA) was added to educe the resulting formazan, and cells were incubated for 15 min at 37. At last, the absorbance of each solution was measured at 570 nm.

### Immunofluorescence

The cardiac tissues in different groups were prepared into frozen sections for immunofluorescence analysis. The 4.5-um frozen section and cardiac fibroblasts were fixed for 20 min in 4% paraformaldehyde (PFA) at room temperature. After fixation, they were blocked with 5% bovine serum albumin (BSA) in Tris Buffered Saline With Tween (TBST) for 1 h, and then incubated with appropriate primary antibodies at 4 °C over night and incubated with second antibodies (Alexa Fluor ®488-conjugated affinipure goat anti-rabbit lgG (H+L), Cy^TM^ 3-conjugated affinipure donkey anti-goat^++^ lgG (H+L), and Alexa Fluor ®488-conjugated affinipure donkey anti-goat++ lgG (H+L) (Jackson ImmunoResearch Laboratories, Inc., PA, USA)) for 1 hour at room temperature. After that, sections and cells were stained with 1.5 μM 2-(4-Amidinophenyl)-6-indolecarbamidine dihydrochloride (DAPI; Sigma, St. Louis, Missouri, USA) for 10 min. The image analysis of frozen sections was performed using a software program (Olympus, Japan). Protein localization of cells was observed and captured with a laser scanning confocal microscope (LSM5, Zeiss, Jena, Thuringia, Germany).

### Statistical analysis

Statistics were performed using GraphPad Prism software (GraphPad Software, Inc., CA, USA). All data was analyzed by SPSS 17.0 software (SPSS Inc, Chicago, Illinois, 2008). All experiments were repeated with at least three batches of cardiac fibroblasts, and qualitatively similar data were obtained in all repetitions. Data were tested with Analysis of Variance (ANOVA) or t-test, with *P* < 0.05 considered significant.

## Results

### Evidence of ANO1 in the neonatal rat cardiac fibroblasts

**Figure 1:**
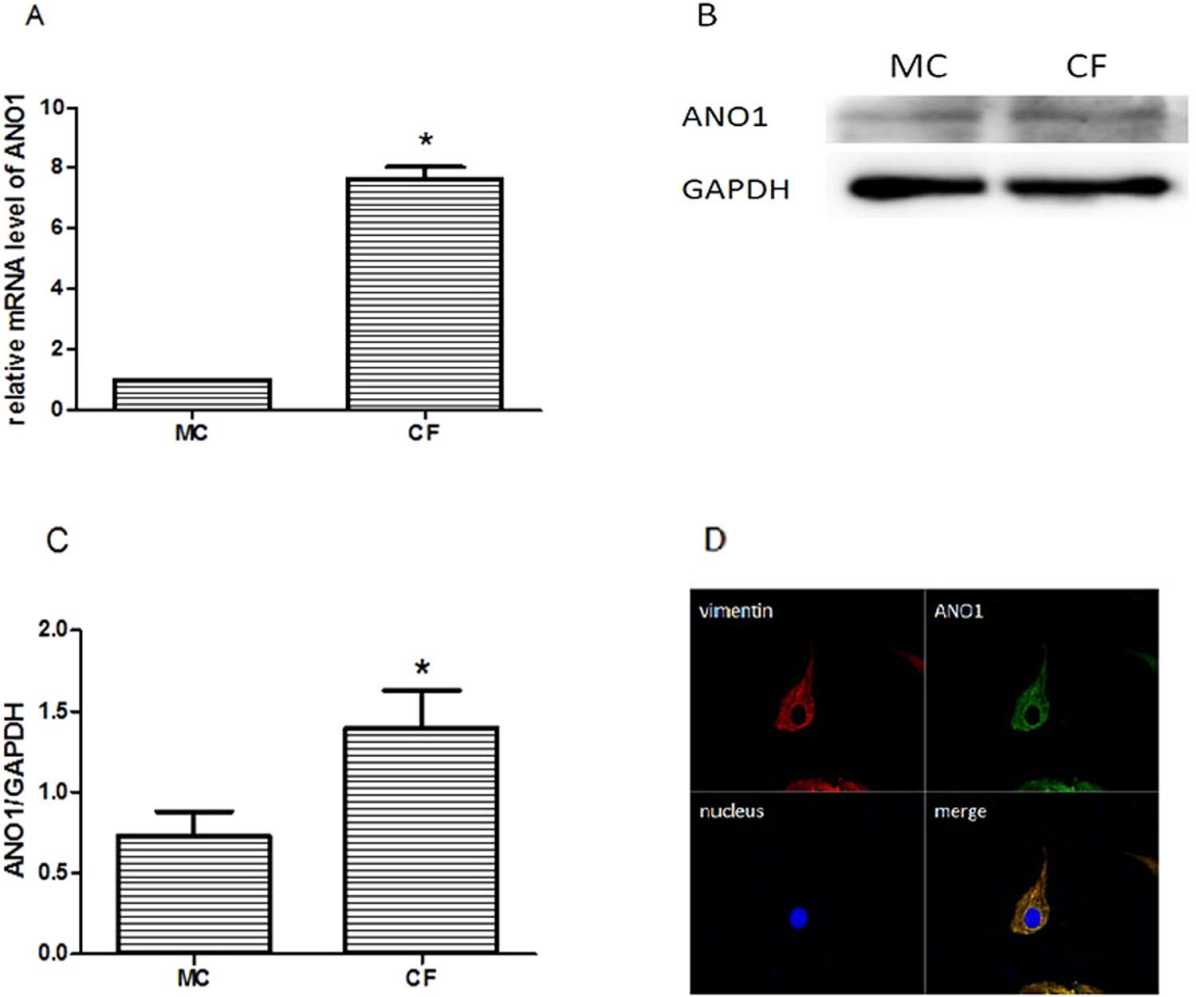
Expression of ANO1 in myocardial cell and cardiac fibroblasts by qRT-PCR (**A**), western bloting (**B**,**C**), and immunofluorescent confocal microscopic analysis (**D**). (**A**) qRT-PCR showed the relative mRNA level of *Ano1* in myocardial cell and cardiac fibroblasts. (**B**,**C**) Western blotting demonstrated the expression of ANO1 protein in myocardial cell and cardiac fibroblasts. (**D**) Immunoflurescent confocal microscopy showed ANO1 protein expression profiles, cardiac fibroblasts were identified by vitemtin staining (red), ANO1 protein was green staining, and nuclei were identified by DAPI staining (blue). MC as myocardial cell, CF as cardiac fibroblasts. *p < 0.05, **p < 0.01.

Cardiac fibroblasts and myocardial cells of neonatal rats were isolated to detect *Ano1* mRNA and protein expression. The expression of *Ano1* mRNA in cardiac fibroblasts was detected by qRT-PCR analysis (Fig. 1A) and ANO1 protein by western blotting analysis (Fig. 1B,C), which were significantly higher than those in myocardial cells. Furthermore we stained cardiac fibroblasts (red), ANO1 protein (green), and nucleus (blue) by immunofluorescent confocal microscopic analysis (Fig. 1D). These results demonstrated ANO1 protein existed in the cardiac fibroblasts of neonatal rats and the expression was higher than that in myocardial cells.

### ANO1 expression increased cardiac fibrosis after hypoxia

**Figure 2:**
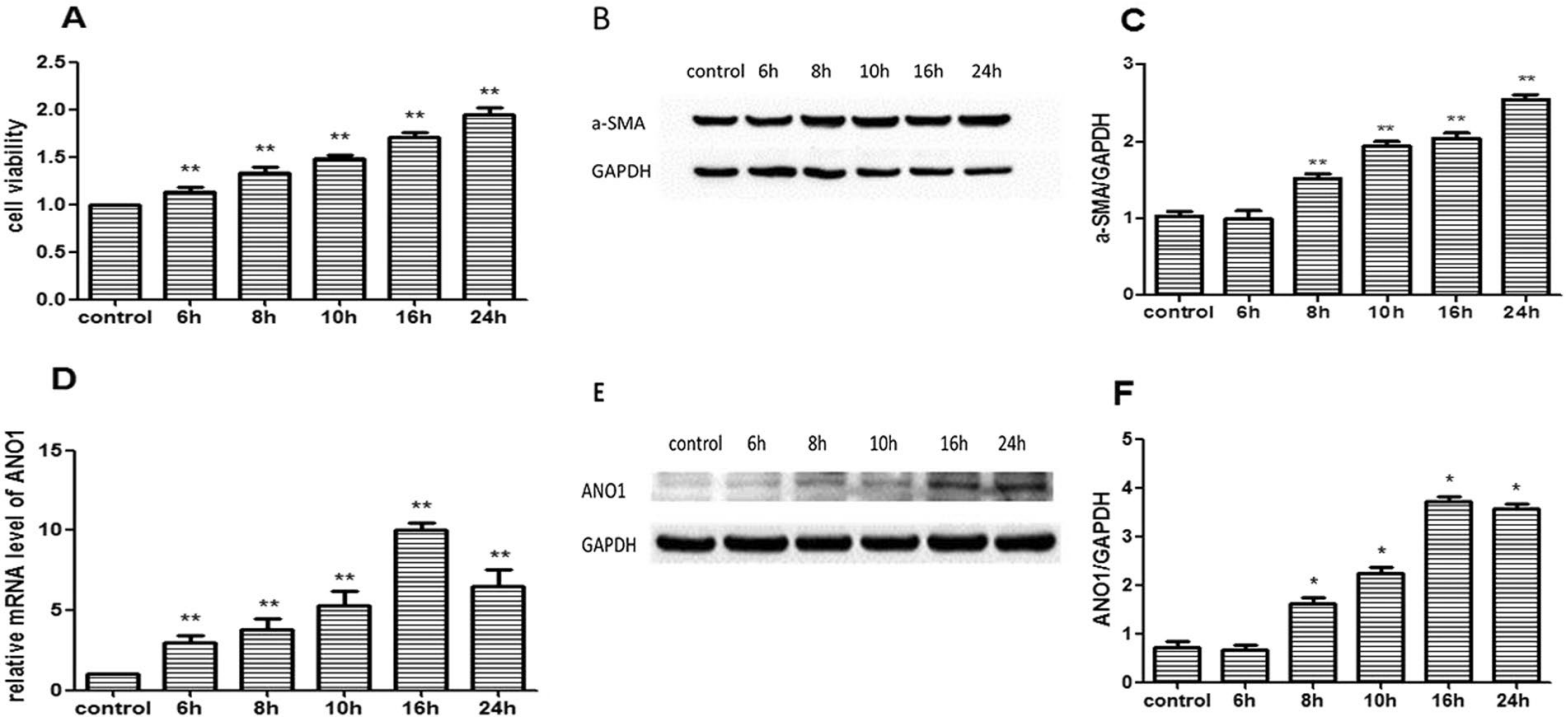
ANO1 expression and indicators expressions of cardiac fibrosis after hypoxia in cardiac fibroblasts. (**A**) MTT analysis showed cell viability growed gradually at 6, 8, 10, 16, and 24 hours after hypoxia. (**B** and **C**) a-SMA expression was gradually increased at 6, 8, 10, 16, and 24 hours after hypoxia compared to untreated group, and peaked at 24 hours by western blotting analysis. (**D**) *Ano1* mRNA expression in cardiac fibroblasts at 6, 8, 10, 16, and 24 hours after hypoxia by qRT-PCR. (**E** and **F**) ANO1 protein expression in cardiac fibroblasts at 6, 8, 10, 16, and 24 hours after hypoxia by western blotting analysis. *p < 0.05, **p < 0.01.

Our previous study^31^ demonstrates that hypoxia promotes the proliferation of cardiac fibroblasts and their offspring myofibroblasts. In this study, MTT analysis verified that cell viability at the point of 6, 8, 10, 16, and 24 hour increased gradually after hypoxia (Fig. 2A). According to western blotting analysis, compared to untreated group, the level of a-SMA (marker of myofibroblasts) at the point of 6, 8, 10, 16, and 24 hourincreased gradually after hypoxia and peaked at the point of 24 hour (Fig. 2B,C). These results demonstrated that cardiac fibroblast activated after hypoxia (e.g. fibroblasts proliferation) developed into myofibroblasts and set off cardiac fibrosis. Then we detected *Ano1* mRNA and protein expression in cardiac fibroblasts 6, 8, 10, 16, and 24 hours after hypoxia by qRT-PCR (Fig. 2D), and western blotting (Fig. 2E,F). The expression of *Ano1* mRNA and protein increased as hypoxia went on in the first 24 hours, and peaked16 hours after hypoxia. These results suggest that ANO1 participates in cardiac fibrosis after cardiac fibroblasts hypoxia.

### Expression of ANO1 increased in myocardial infraction (MI) mice

**Figure 3:**
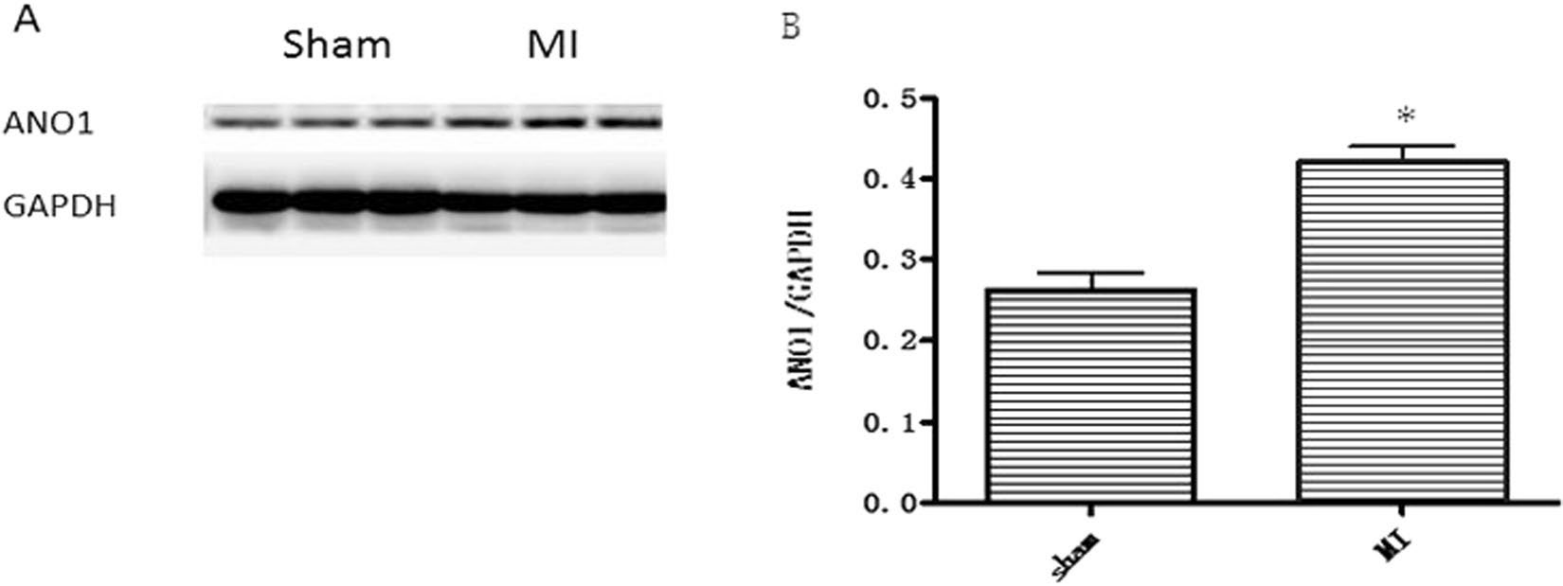
ANO1 is expressed in mouse myocardium and increased in infarcted zone post MI. (**A**) Protein extracts were prepared for immunoblot analysis against ANO1. The blots against GAPDH served as loading controls. (**B**) Quantitative analysis of ANO1. ∗p < 0.05 versus sham. *n* = 6 per group.

Western Blot was used to evaluate the expression of ANO1 in MI mice. ANO1 increased more significantly in MI group compared with the sham group (Fig. 3A,B).

### Overexpression of ANO1 reduced the indicators of cardiac fibrosis after hypoxia

**Figure 4:**
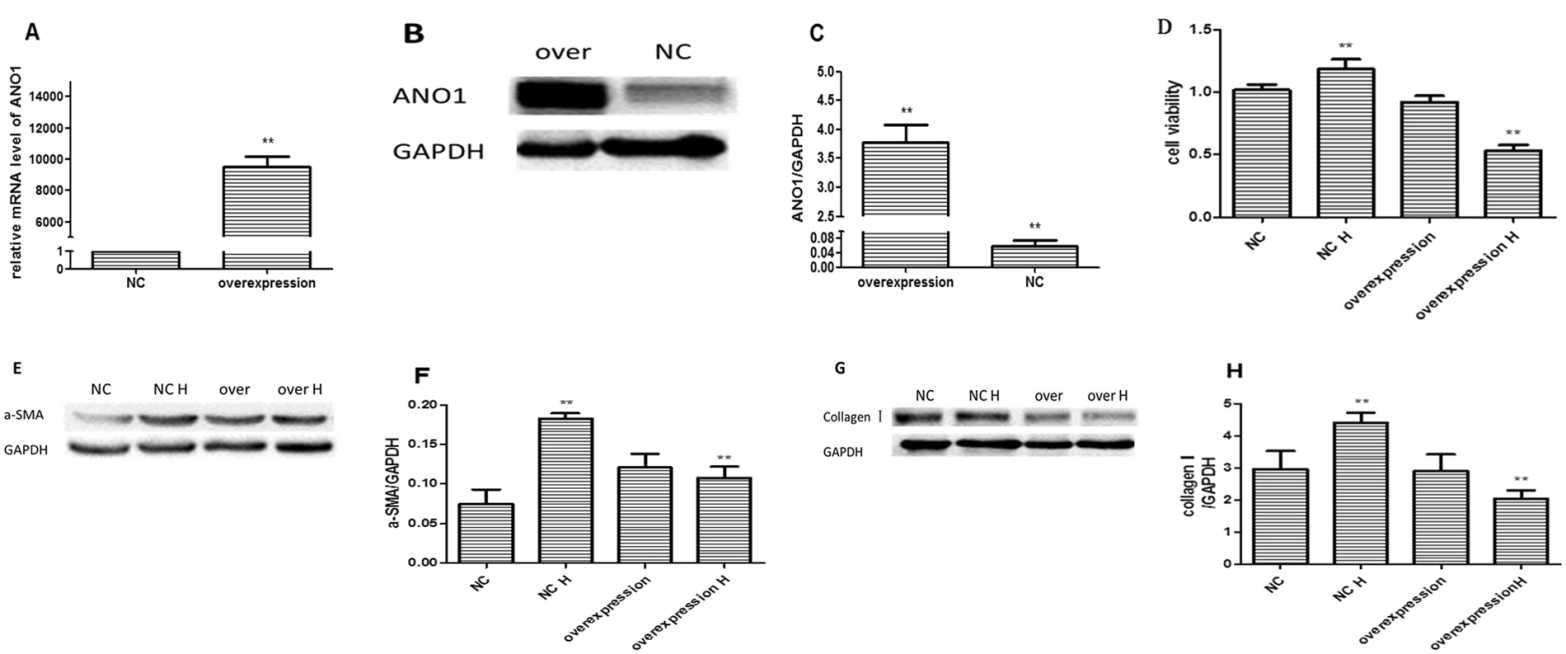
Overexpression of ANO1 reduced the indicators of cardiac fibrosis after hypoxia. (**A**) *Ano1* mRNA was dramatically increased in ANO1 overexpression group comparing to negative control (NC) group by qRT-PCR. (**B** and **C**) ANO1 protein expression were dramatically increased in ANO1 overexpression group comparing to NC group by western blotting assay. (**D**) MTT analysis showed that cell viability elevated in overexpression ANO1 + hypoxia 16 hours (overexpression H) group comparing to overexpression ANO1 (over H) group and negative control (NC) + hypoxia 10 hours (NC H) group. (**E** and **F**) Expression of a-SMA protein was reduced in overexpression H group comparing to overexpression group and NC H group by western blotting. (**G** and **H**) Expression of collagen I protein was reduced in overexpression H group comparing to overexpression group and NC H group by western blotting. *p < 0.05, **p < 0.01.

To investigate the function of ANO1 in cardiac fibroblasts, we transduced adenovirus-encoding *Ano1* (Ad-GFP-ANO1) or GFP (Ad-GFP) into cardiac fibroblasts. According to qRT-PCR (Fig. 4A) and western blotting, *Ano1* mRNA and protein expression increased dramatically in *Ano1* over-expression group compared with negative control group (Fig. 4B,C), respectively. These results proved that ANO1 expression model was well constructed. Secondly, cell viability decresed in overexpression ANO1 + hypoxia 16 hours (overexpression H) group compared with overexpression ANO1 (overexpression) group and negative control (NC) + hypoxia 16 hours (NC H) group (Fig. 4D). This result demonstrated that ANO1 over-expression inhibited the proliferation of cardiac fibroblasts after hypoxia. Thirdly, according to Western blotting, a-SMA (Fig. 4E,F) and collagen I (Fig. 4G,H) protein levels were reduced in over-expression H group compared with over-expression group and NC H group. We summarized that the over-expression of ANO1 inhibited cardiac fibrosis after hypoxia.

### ANO1 alleviated fibrosis after MI

**Figure 5:**
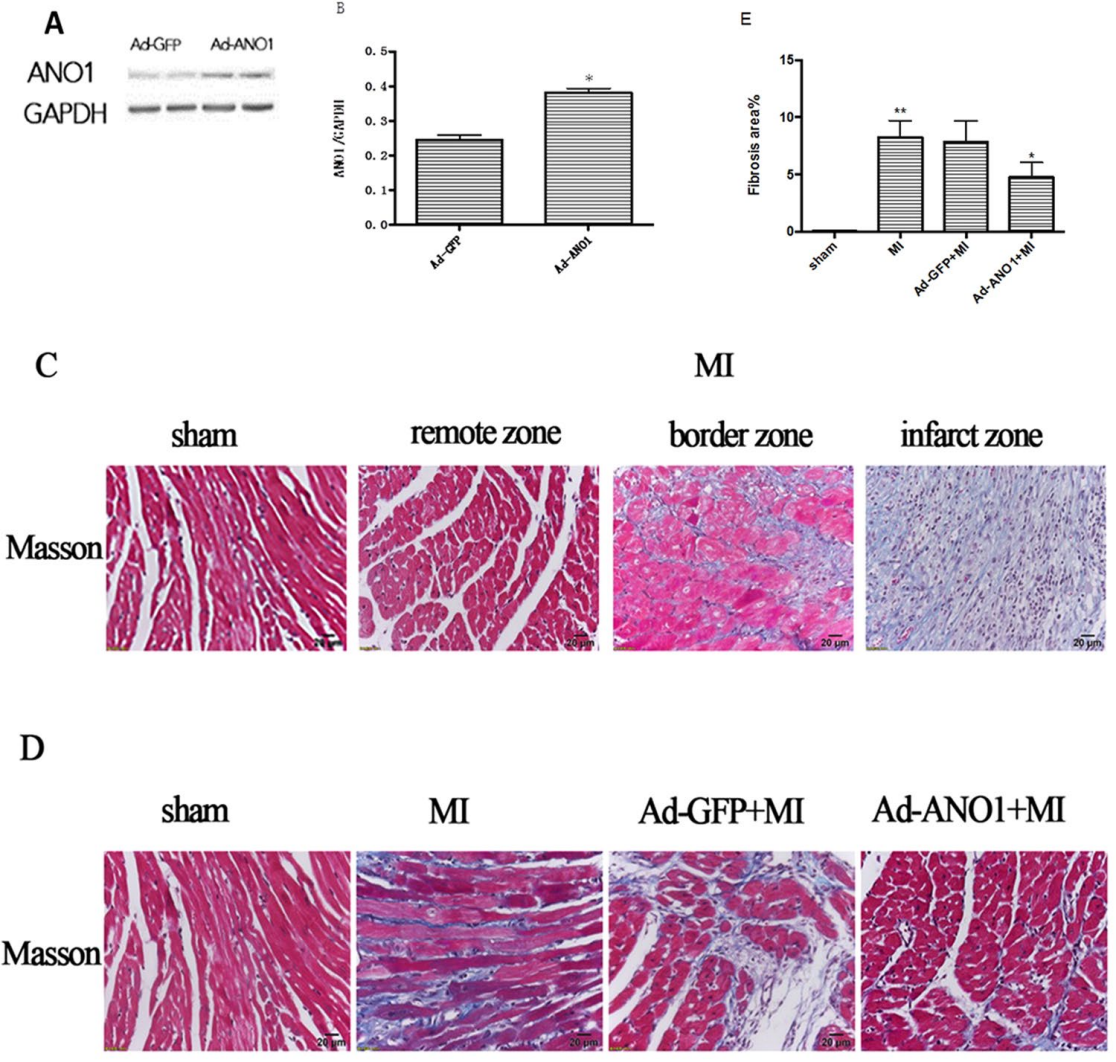
Overexpression of ANO1 attenuates cardiac fibrosis post-MI. (**A**)Western blot of ANO1 expression levels in Ad-ANO1+MI and Ad-GFP+MI group. The blots of GAPDH serves as loading controls. (**B**) Quantitative analysis of ANO1. (**C**) Representative Masson’s trichrome staining in sham group and remote zone, border zone, infarct zone of MI group. Blue staining indicates connective tissue. Scale bars represent 20 μm. (**D**) Representative Masson’s trichrome staining in sham, MI, Ad-ANO1+MI and Ad-GFP+MI. Scale bars represent 20 μm. (**E**) The quantification of fibrosis. *P < 0.05 versus MI or Ad-GFP+MI.**P < 0.01 versus sham. *n* = 6 per group.

To reveal the role of TMEM16A in fibrosis after MI, Ad-GFP or Ad-ANO1 was injected into the left ventricular (LV) wall immediately after MI. Western blot showed the protein of ANO1 increased significantly in mice with Ad-ANO1 compared to mice with Ad-GFP, which confirms the successful gene transfer (Fig. 5A,B). We examined myocardial fibrosis using Masson’s Trichrome staining. Obvious fibrosis was observed in the infarcted margin (Fig. 5C). Fibrosis decreased significantly in the Ad-ANO1 group compared with MI and Ad-GFP group (Fig. 5D,E).

### ANO1 inhibited myofibroblast differentiation *in vivo*

**Figure 6:**
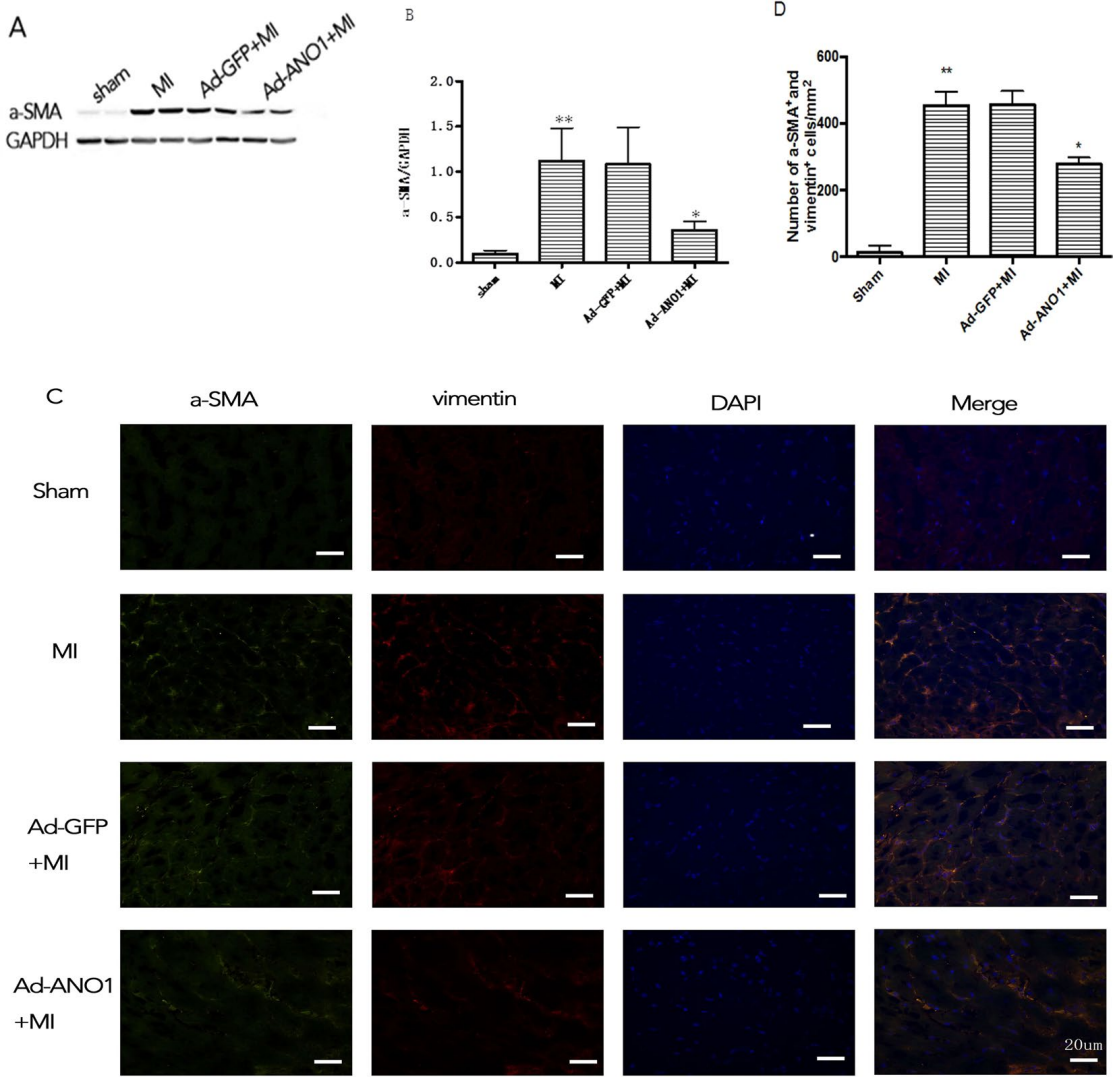
ANO1 inhibited myofibroblast differentiation in vivo. (**A**) Western blot of a-SMA protein expression. GAPDH was used as loading controls. (**B**) Quantitative analysis of a-SMA. (**C**)Representative immunofluorescence image of a-SMA and vimentin, green, a-SMA; red,vimentin; blue, nuclei. Scale bars represent 20 μm. (**D**) The quantification of number of a-SMA and vimentin positivestaining cells per mm2. *p < 0.05 versus MI or Ad-GFP+MI,**p < 0.01 versus sham (n = 6).

Myofibroblast is responsible for interstitial matrix and consequent left ventricle (LV) deformation caused with fibrosis. We evaluated the effect of ANO1 on cardiac myofibroblast differentiation in MI modle. Western blot showed ANO1 reduced the expression of a-SMA significantly (Fig. 6A,B). Immunofluorescence analysis also revealed that ANO1 reduced the number of a-SMA and vimentin positive staining cells in the bordering zone (Fig. 6C,D).

### ANO1 inhibits cardiac fibrosis via TGF-β/smad3 pathway

**Figure 7:**
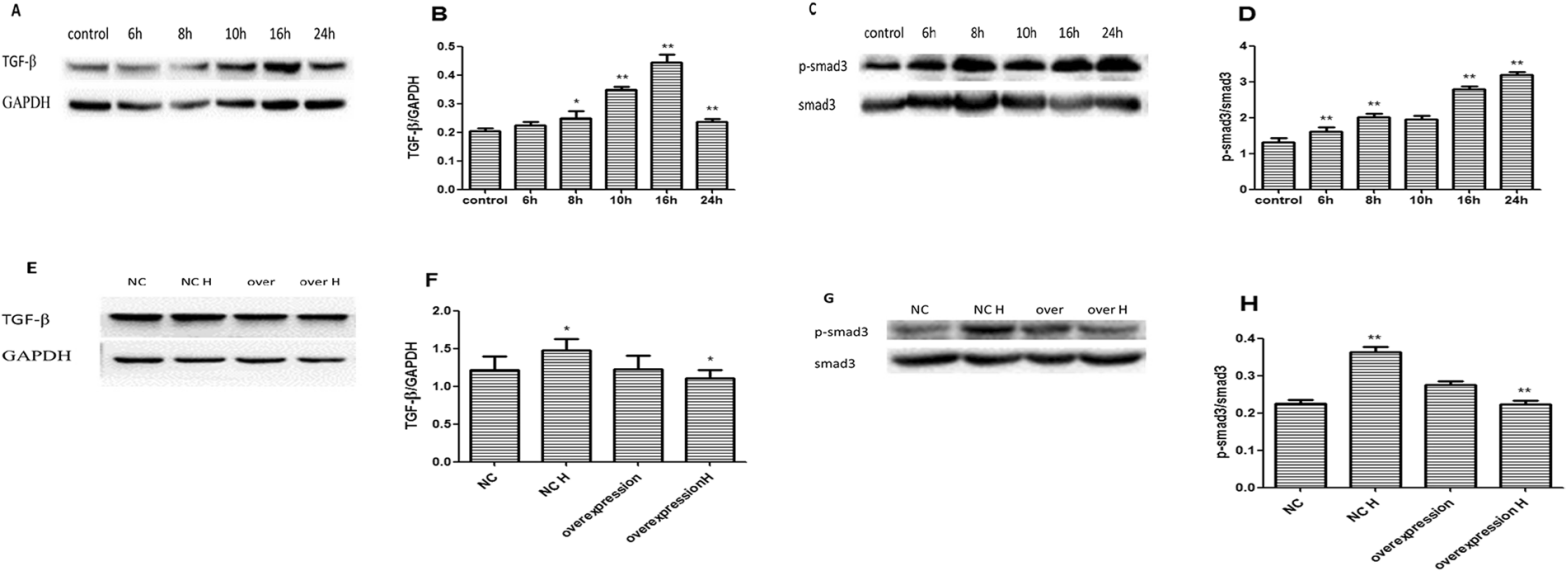
ANO1 inhibits cardiac fibrosis via TGF-β/smad3 pathway. (**A** and **B**) At 6, 8, 10, 16, and 24 hours after hypoxia, TGF-β protein levelwas gradually increased compared to untreated group, peaked at 16 and 24 hours by western blotting analysis. (**C** and **D**) At 6, 8, 10, 16, and 24 hours after hypoxia, p-smad3protein levelwas gradually increased compared to untreated group, peaked at 16 and 24 hours by western blotting analysis. (**E**) and (**F**) After cardiac fibroblasts were tranfected by Ad-GFP-ANO1 and exposed in the present or absent hypoxia, TGF-β protein level was decreased in overexpression H group comparing to overexpression group or negative control hypoxia (NC H) group by western blotting. (**G** and **H**) After cardiac fibroblast were tranfected by Ad-GFP-ANO1 and exposed in the present or absent hypoxia, TGF-β protein level was decreased in overexpression H group comparing to overexpression group or negative control hypoxia (NC H) group by western blotting. *p < 0.05, **p < 0.01.

To understand how ANO1 regulated cardiac fibrosis in cardiac fibroblasts, we detected the expression of fibrosis in cardiac fibroblasts infected with Ad-GFP-ANO1. According to western blotting analysis, protein levels at the point of 6, 8, 10, 16, and 24 hours after hypoxia, TGF-β (Fig. 7A,B) and p-smad3 (Fig. 7C,D) increased gradually and peaked at 16 and 24 hours compared to untreated group. The protein expression of TGF-β and p-smad3 were consistent with ANO1 and fibrosis indicators expression after hypoxia above. Moreover, after the transfection of cardiac fibroblasts with Ad-ANO1-GFP and their 16 hours exposure to hypoxia, we found that TGF-β (Fig. 7E,F) and p-samd3 (Fig. 7G,H) protein levels decreased in group of over-expression H group compared with over-expression group or negative control(NC) H group by western blotting. These data indicate that ANO1 inhibits cardiac fibrosis via deregulating TGF-β/smad3 pathway.

## Discussion

ANO1 acts as a mediator of Cl^−^ secretion in secretory epithelia, a heat sensor in neurons, a controler of smooth muscle tone, and a trigger in tumorigenesis. Since ANO1 was first confirmed as a molecular basis of CaCCs in 2008, it has been repeatedly found in proliferating cells. However, in our experiment, we show for the first time that ANO1 exists in rat cardiac fibroblasts and maneovers cardiac fibrosis after cell hypoxia or MI via TGF-β/smad3 pathway, and that ANO1 over-expression in cardiac fibroblasts or other heart tissues promotes cardiac fibrosis. All together, ANO1 is a potential therapeutic target in cardiac fibrosis after MI. Nevertheless, considering TGF-β/smad3 pathway in fibrosis, we cannot detect other fibrosis signaling pathway. Furthermore, we cannot study the effect of ANO1 knockdown on fibrosis. Further experiments are needed to clarify these points.

In this experiment, *Ano1* mRNA and protein expressionin cardiac fibroblasts of neonatal rats increased after hypoxia. In 2008, three research groups verifies that ANO1 is an essential factor in CaCCs. Some other studies demonstrate that Cl^−^ channels regulate the proliferation of many cells^34, 35^. ANO1 expression is also down-regulated in smooth muscle cells isolated from the basilar arteries of hypertensive rats with chronic hypoxia^36^. In contrast, ANO1 expression is up-regulated in pulmonary arterial smooth muscle cells of rats with chronic hypoxia^37^. Therefore, the expression of ANO1 after hypoxia is decided by the type of cells. So one gene functions differently in different cell lines. This experiment first found that ANO1 expression increased in cardiac fibroblasts after hypoxia. However, what brings this increase and which of ANO1 and cardiac fibrosis comes first should be answered by further study. We suggest that ANO1 evokes the protective response to hypoxia and subsequent cardiac fibrosis.

To investigate the role of ANO1 in cardiac fibrosis, we examined the effect of ANO1 over-expression on proliferation and differentiation of rat cardiac fibroblasts. Our data indicated that ANO1 over-expression inhibited the proliferation and differentiation of cardiac fibroblasts after cell hypoxia. Other groups have verified that Cl^−^ channels including CaCCs which molecular basis is ANO1 regulated cell proliferation and cell cycle progression in a variety of cell types34,35. Moreover, ANO1 impedes cell proliferation by arresting the G0/G1 phase of cell cycle and reducing cyclin D1 and cyclin E expression^36^. Our animal experiment showed that over-expression of ANO1 largely inhibited cardiac fibrosis after MI. These results suggest that over-expression of ANO1 can improve cardiac fibrosis after MI.

However, how ANO1 regulates cardiac fibroblasts differentiation and proliferation remains unanswered. We studied the relationship between ANO1 and cardiac fibrosis signaling pathway, and the relationship between ANO1 and TGF-β/smad3 pathway. Over-expression ANO1 inhibited TGF-β/smad3 pathway and subsequent cardiac fibrosis. Thus, we concluded that ANO1 takes part in cardiac fibrosis, probably through TGF-β/smad3 pathway. Nevertheless we cannot detect other fibrosis signaling pathway and the effect of ANO1knockdown on fibrosis. Further experiments are needed.

Taken together, our studies show that ANO1 participates in cardiac fibrosis via TGF-β/smad3 pathway. For the first time, our data provide a deep insight into the role of ANO1 in MI. This finding helpsdevelop a therapeutic target with ANO1 for cardiac remodeling after MI.

## References

[1] Zarrabi, K. *et al*. The Comparison Between Two Surgical Methods for Left Internal Mammary Artery (LIMA) Anastomosis on Left Anterior Descending (LAD) Artery in Patients with Severe Diffuse Lesions: Short to Mid-Term Results. *Acta Medica Iranica.* **53**(6), 369–372 (2015). 26069175

[2] Spiliopoulos S, Koerfer R, Tenderich G (2016). Acute myocardial infarction complicated by cardiogenic shock: results of primary percutaneous coronary interventions are insufficient. Eur J Cardiothorac Surg..

[3] Wu D (2015). CTRP3 attenuates post-infarct cardiac fibrosis by targeting Smad3 activation and inhibiting myofibroblast differentiation. J Mol Med..

[4] Sarrazy V (2014). Integrins alphavbeta5 and alphavbeta3 promote latent TGFbeta1 activation by human cardiac fibroblast contraction. Cardiovasc Res..

[5] Schuetze KB, McKinsey TA, Long CS (2014). Targeting cardiac fibroblasts to treat fibrosis of the heart: focus on HDACs. J Mol Cell Cardiol..

[6] Mao Q, Lin CX, Liang XL, Gao JS, Xu B (2013). Mesenchymal stem cells overexpressing integrin-linked kinase attenuate cardiac fibroblast proliferation and collagen synthesis through paracrine actions. Mol Med Rep..

[7] Wang L (2014). The impact of 1,25-dihydroxyvitamin D3 on the expression of connective tissue growth factor and transforming growth factor-beta (1) in the myocardium of rats with diabetes. Diabetes Res Clin Pract..

[8] Dean RG (2005). Connective tissue growth factor and cardiac fibrosis after myocardial infarction. J Histochem Cytochem..

[9] Gaspard GJ, MacLean J, Rioux D, Pasumarthi KB (2014). A novel beta-adrenergic response element regulates both basal and agonist-induced expression of cyclin-dependent kinase 1 gene in cardiac fibroblasts. Am J Physiol Cell Physiol..

[10] Berk BC, Fujiwara K, Lehoux S (2007). ECM remodeling in hypertensive heart disease. J Clin Invest..

[11] Hartzell C, Putzier I, Arreola J (2005). Calcium-activated chloride channels. Annu Rev Physiol..

[12] Jin X, Shah S, Du X, Zhang H, Gamper N (2016). Activation of Ca(2+) -activated Cl(−) channel ANO1 by localized Ca(2+) signals. J Physiol..

[13] Yang YD (2008). TMEM16A confers receptor-activated calcium-dependent chloride conductance. Nature..

[14] Schroeder BC, Cheng T, Jan YN, Jan LY (2008). Expression cloning of TMEM16A as a calcium-activated chloride channel subunit. Cell..

[15] Caputo A (2008). TMEM16A, a membrane protein associated with calcium-dependent chloride channel activity. Science..

[16] Ayoub C (2010). ANO1 amplification and expression in HNSCC with a high propensity for future distant metastasis and its functions in HNSCC cell lines. British journal of cancer.

[17] Liu W, Lu M, Liu B, Huang Y, Wang K (2012). Inhibition of Ca(2+)-activated Cl(−) channel ANO1/TMEM16A expression suppresses tumor growth and invasiveness in human prostate carcinoma. Cancer letters..

[18] Berglund E (2014). Functional role of the Ca^2+^-activated Cl^−^ channel DOG1/TMEM16A in gastrointestinal stromal tumor cells. Exp Cell Res..

[19] Gomez-Pinilla PJ (2009). Ano1 is a selective marker of interstitial cells of Cajal in the human and mouse gastrointestinal tract. Am J Physiol Gastrointest Liver Physiol..

[20] Komatsu Y (2006). TAOS1, a novel marker for advanced esophageal squamous cell carcinoma. Anticancer Res..

[21] Duvvuri U (2012). TMEM16A induces MAPK and contributes directly to tumorigenesis and cancer progression. Cancer Res..

[22] Sui Y (2014). Inhibition of TMEM16A expression suppresses growth and invasion in human colorectal cancer cells. PLoS One..

[23] Liu J, Liu Y, Ren Y, Kang L, Zhang L (2014). Transmembrane protein with unknown function 16A overexpression promotes glioma formation through the nuclear factor-kappaB signaling pathway. Mol Med Rep..

[24] El Chemaly A (2014). ANO1 contributes to angiotensin-II-activated Ca-dependent Cl current in human atrial fibroblasts. J Mol Cell Cardiol..

[25] Pickup M, Novitskiy S, Moses HL (2013). The roles of TGFβ in the tumour microenvironment. Nat Rev Cancer.

[26] Siegel PM, Massagué J (2003). Cytostatic and apoptotic actions of TGF-beta in homeostasis and cancer. Nat Rev Cancer.

[27] Bierie B, Moses HL (2006). Tumour microenvironment: TGFbeta: the molecular Jekyll and Hyde of cancer. Nat Rev Cancer.

[28] Macias-Silva M (1996). MADR2 is a substrate of the TGFbeta receptor and its phosphorylation is required for nuclear accumulation and signaling. Cell..

[29] Zhu J (2002). beta8 integrins are required for vascular morphogenesis in mouse embryos. Development..

[30] Olman MA (2009). Beyond TGF-beta: a prostaglandin promotes fibrosis. Nat Med..

[31] Gao Y, Chu M, Hong J, Shang JP, Xu D (2014). Hypoxia induces cardiac fibroblast proliferation and phenotypic switch: a role for caveolae and caveolin-1/PTEN mediated pathway. J Thorac Dis..

[32] Qu, X. *et al*. Expression signature of lncRNAs and their potential roles in cardiac fibrosis of post-infarct mice. *Biosci Rep*. **36**, pii: e00337 (2016). 10.1042/BSR20150278 PMC5293569 27129287

[33] Lin S (2016). HSP27 Alleviates Cardiac Aging in Mice via a Mechanism Involving Antioxidation and Mitophagy Activation. Oxid Med Cell Longev..

[34] Guan YY, Wang GL, Zhou JG (2006). The ClC-3 Cl^−^ channel in cell volume regulation,proliferation and apoptosis in vascular smooth muscle cells. TrendsPharmacol Sci..

[35] Habela CW, Olsen ML, Sontheimer H (2008). ClC3 is a critical regulator of the cell cycle in normal and malignant glial cells. J Neurosci..

[36] Wang M (2012). Downregulation of TMEM16A calcium-activated chloride channel contributes to cerebrovascular remodeling during hypertension by promoting basilar smooth muscle cell proliferation. Circulation..

[37] Sun H, Xia Y, Paudel O, Yang XR, Sham JS (2012). Chronic hypoxia-induced upregulation of Ca^2+^-activated Cl^−^ channel in pulmonary arterial myocytes: a mechanism contributing to enhanced vasoreactivity. J Physiol..

